# Apple Cider Vinegar-Induced Hepatotoxicity: A Rare Case Report

**DOI:** 10.1093/omcr/omaf097

**Published:** 2025-07-14

**Authors:** Celina R Andonie, Saja I AbuGhannam, Shadi M A Ruzayqat

**Affiliations:** Faculty of Medicine, Al Quds University, University Street, Abu Dis, Jerusalem, P.O. Box 89, Postal Code P144, Palestine; Faculty of Medicine, Al Quds University, University Street, Abu Dis, Jerusalem, P.O. Box 89, Postal Code P144, Palestine; General Surgery Department, Al-Ahli Hospital, Be'er Almahjar, Hebron, Palestine

**Keywords:** drug induced liver injury (DILI), apple cider vinegar, vinegar-induced liver injury, traditional substances, herbal remedies

## Abstract

Apple cider vinegar is a ubiquitous ingredient that has been used for many years. There are not many reports in the literature of idiosyncratic drug-induced liver damage linked to apple cider vinegar in people. As far as we are aware, liver damage is an uncommon side effect of long-term apple cider vinegar use. We describe the case of a 60-year-old man who had a long history of vinegar consumption and now had vinegar-induced hepatotoxicity. After the patient stopped using vinegar, his condition improved. This case emphasizes how important it is to take into account conventional substances like vinegar as possible causes of liver damage.

## Introduction

Apple cider vinegar (ACV) is a clear, colorless, sour liquid composed of water and 3%–5% acetic acid, primarily produced with the help of acetic acid bacteria through an oxidation process. ACV has multiple uses, including in cooking, medicine, and cleaning. ACV is well-known for its potential health benefits, such as its anticancer properties and reducing blood glucose levels in patients with type 2 diabetes [1]. Various reports have demonstrated that long-term consumption of vinegar decreases LDL-c and increases HDL-c in noncontrolled rat experiments [2].

Since the liver is primarily responsible for metabolizing most medications and substances, it is highly vulnerable to drug-induced liver injury (DILI). DILI can present as either an acute or chronic response and may be caused by nearly all classes of medications, as well as by various traditional substances (e.g. ACV) and herbal remedies [3]. The hepatotoxicity of drugs occurs through two major mechanisms: dose-dependent reactions and idiosyncratic reactions [4].

Several cases of highly concentrated acetic acid-induced hepatotoxicity have been reported. In a fatal case described by Kamijo et al., a 42-year-old woman developed hemolysis, metabolic acidosis, disseminated intravascular coagulation (DIC), and hepatic failure within 45 min of ingesting 200 ml of 90% acetic acid, resulting in massive periportal hepatic necrosis and eventual death 39 h post-ingestion. In another case reported by Gerhartz, a patient died eight days after ingesting 200 ml of 60% acetic acid, with autopsy findings revealing periportal hepatic necrosis, inflammatory cell infiltration, and signs of regeneration [5]. A third case by Klunklin described a patient who ingested 30 ml of glacial acetic acid and showed hepatocyte swelling, periportal inflammation, and hyaline degeneration on liver biopsy [6]. Lastly, Kawamata et al. reported a case of acute liver dysfunction following rectal administration of 50 ml of 9% acetic acid, although no biopsy was performed [7]. The uniqueness of our case is that the patient was exposed to a lower concentration of acetic acid for a long period of time.

## Case report

A 60-year-old man complained of acute pain in the upper right quadrant region. For a week, it was associated with anorexia, clay-colored stools, tea-colored urine, and jaundice. No history of bloody stools, fever, itching, vomiting, weight loss, or altered bowel habits. Clinical signs of autoimmune disorders, including joint discomfort, photosensitivity, dry eyes and mouth, and oral ulcers, were absent.

The patient denied any history of blood transfusions, high-risk sexual behavior, alcohol or illicit drug use, and over-the-counter medication use. He also had no history of inherited liver disorders, or neuropsychiatric conditions. His medical history was notable for hypertension, hyperlipidemia, and mild fatty liver, with no significant surgical history, and he had an allergy to penicillin. However, the patient reported drinking about 30 cc of ACV three times weekly after meals for the past four years due to its reputed benefits for lowering lipids, particularly in relation to his fatty liver.

On admission, the patient was conscious, oriented, and alert, vitally stable with no signs of hepatic encephalopathy. His BMI was normal. There were no Kayser-Fleischer rings or xanthelasma, and the fundus exam was normal. No rashes or lymphadenopathy were noted, and there were no stigmata of chronic liver disease or chronic alcohol use. However, he was clinically jaundiced, exhibiting scleral icterus, sublingual jaundice, and diffuse yellowish discoloration of the skin. He had tenderness in the right upper quadrant. The rest of the cardiovascular, respiratory, and neurological examinations were normal.

**Figure 1:**
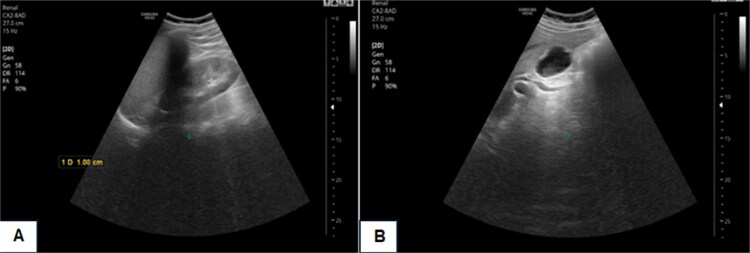
Abdominal ultrasound showing a mild liver fatty infiltration. The gallbladder was normal in size with a small sludge (white arrow in image B) measuring 0.8 × 0.4 cm.

His blood investigations, including CBC, renal function tests, and inflammatory markers, were within normal limits. Serology was negative for hepatitis B and C but positive for hepatitis A IgG antibodies. EBV, CMV and HIV serology were negative. An extensive autoimmune workup, including ANA, ASMA, and LKM-1, was negative. CEA and CA 19–9 levels were unremarkable. An abdominal ultrasound was performed, revealing the following findings: the liver was at the upper limit of normal size with mild fatty infiltration. The gallbladder was normal in size with normal wall thickness, containing a small sludge measuring 0.8 x 0.4 cm. Mild dilatation of the common bile duct (CBD) was noted, measuring 8.5 mm in diameter, along with mild thickening of the CBD wall and increased echogenicity (Fig. 1).

The liver function test showed a hepatocellular pattern of liver injury. The patient was admitted to the surgical ward and started on rehydration therapy and IV antibiotics. The biochemical parameters during his hospital stay (day 1 till day 5), and outpatient follow-up are presented in Table 1.

**Table 1:** Biochemical parameters during hospital stay and follow up.

Biochemical parameters	Reference value	Day 1 (Day of admission)	Day 2	Day 3	Day 5 (Day of discharge)	Day 11	Day 18	Day 25	Day 53	Day 95
Alanine transferase (U/l)	(10–41)	1792	1561	1540	1152	813	953	778	794	324
Aspartate transferase (U/l)	(0–40)	1168	1054	1170	904	862	775	551	509	201
Alkaline Phosphatase (U/l)	(40–130)	190	165	166	137	139	161	136	121	112
Gamma GT (U/l)	(10–71)	158	133	126	114	-	-	-	213	92
Total bilirubin	(0–1.2)	17	17.2	21	21	9.3	5.1	4.4	2.6	1.2
Direct bilirubin	(0–0.2)	-	-	16.4	17	7.5	4.4	3.8	-	-

**Figure 2:**
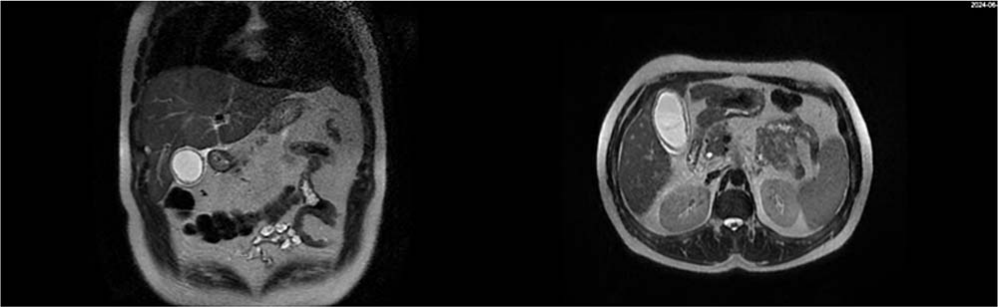
MRI showing wall thickening of the gallbladder, fluid around the gallbladder and a normal diameter of the CBD, as well as the rest of the biliary tree.

**Figure 3:**
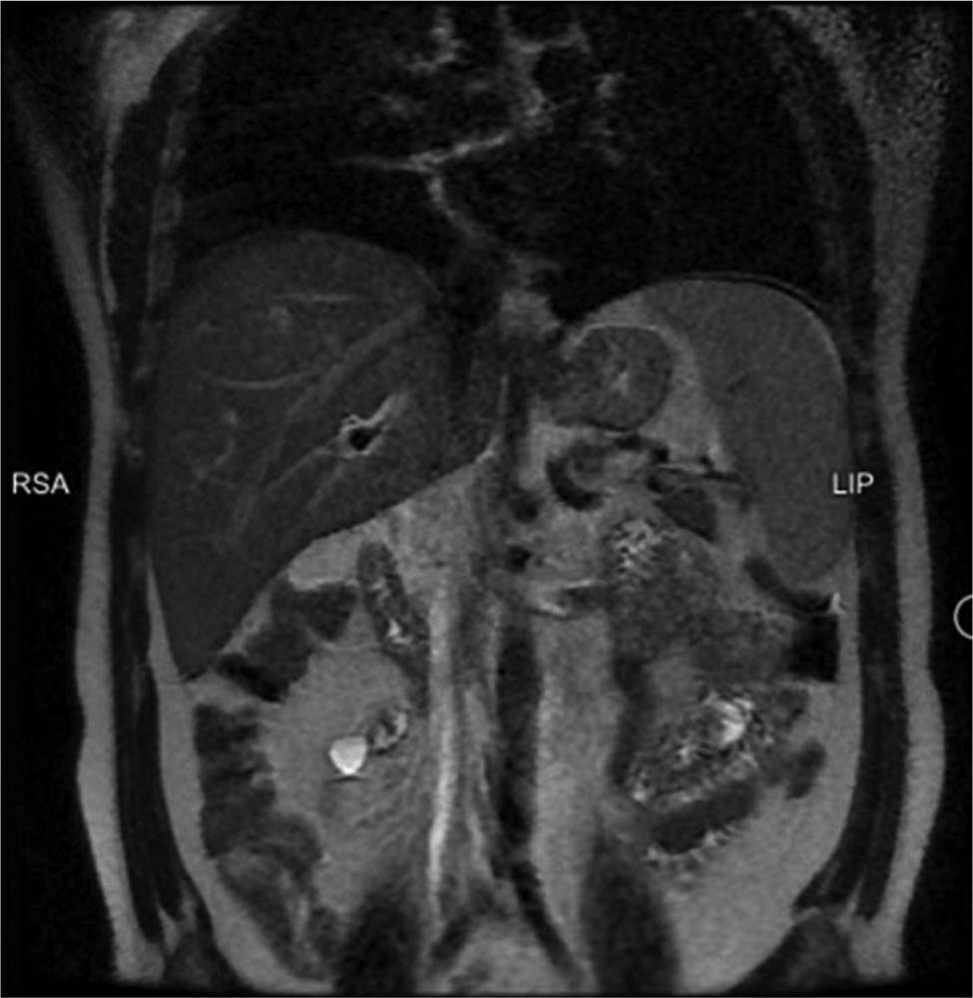
Coronal MRI showing irregularity of the liver border indicating early stage of cirrhosis (black arrowhead).

The patient underwent an ERCP on the second day to rule out other potential causes of liver injury including biliary obstruction or cholestasis. It was performed due to the suspicion of a biliary component to the live injury, as the non-invasive tests did not provide a clear cause. ERCP showed a normal CBD with no obstruction, and a stent was placed. This helped to narrow down the potential causes of liver injury. MRI scan was performed, revealing wall thickening of the gallbladder, fluid around the gallbladder, and a normal diameter of the CBD, as well as the rest of the biliary tree (Figs 2 and 3).

**Figure 4:**
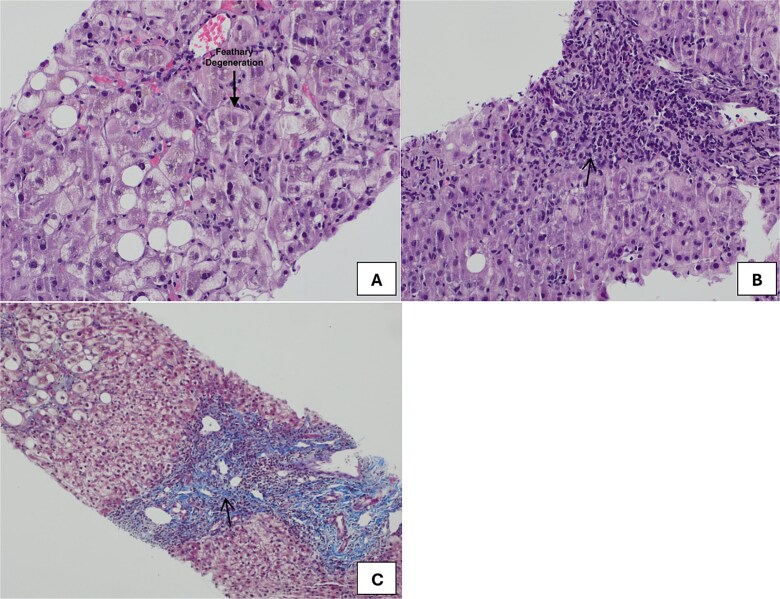
(A) Core liver biopsy demonstrating hepatocyte ballooning and feathery changes (black arrow) with no evidence of confluent necrosis, granulomas, or malignancy. (B) the portal tracts were expanded by mixed inflammatory cells (black arrow), eosinophils, and a few neutrophils with mild bile ductular injury. (C): Masson trichrome special stain showing portal, periportal and bridging fibrosis (black arrow).

However, the patient showed no improvement; and due to diagnosis uncertainty, an ultrasound-guided percutaneous liver core biopsy was obtained. The pathology results indicated cores of hepatic tissue with marked parenchymal and canalicular cholestasis. The hepatocytes showed ballooning and feathery degeneration. The portal tracts were expanded by mixed inflammatory cells, eosinophils, and a few neutrophils with mild bile ductular injury. Foci of interface and lobular activity were noted. Masson trichrome special stain revealed portal, periportal, and bridging fibrosis. There is no evidence of alpha-1 antitrypsin globules by PSA/PSAID special stain. No evidence of iron deposition was found by Perl's special stain. No evidence of confluent necrosis, granulomas, or malignancy. Thus, the final diagnosis based on the liver core biopsy was hepatocellular hepatitis with fibrosis stage 2 (Fig. 4).

The history revealed continuous consumption of a significant amount of ACV over a four-year period, which led to a strong suspicion of vinegar-induced liver injury. The patient was discharged on day 5, prescribed ursodeoxycholic acid 300 mg twice daily, and clearly instructed to completely stop consuming vinegar. Six days later (day 11), he showed gradual improvement in his bilirubin levels and liver function tests. During follow-up visits at the outpatient clinic, it was observed that the yellowish discoloration of his skin and eyes had decreased, along with improvements in his laboratory parameters, indicating ongoing recovery over time, as shown in (Table 1). However, a repeat liver biopsy to confirm improvement in fibrosis after discontinuing ACV ingestion was not performed because the patient refused to consent due to the high cost of the procedure, as he did not have insurance coverage.

## Discussion

ACV is widely used due to its well-known health benefits [1]. However, there is limited information in the literature regarding the correlation between idiosyncratic DILI and the chronic consumption of ACV. This case highlights the importance of considering vinegar and other traditional therapies as potential causes of hepatotoxicity.

DILI is a challenging condition in the clinical setting; it is mainly a diagnosis of exclusion. Although rare in the general population, DILI accounts for most of the cases of acute liver failure, with a mortality rate as high as 50% [8]. The current best practice diagnosis for DILI involves a mix of high clinical suspicion, together with a thorough history of risk factors and timelines, along with comprehensive hepatological investigations. This approach is supported by the international Roussel Uclaf Causality Assessment Method (RUCAM) criteria, considered to be one of the important diagnostic algorithms for DILI [9]. Idiosyncratic DILI generally occurs in an unpredictable way, with the latency period ranging from several weeks to several months [10].

The pattern of liver injury in idiosyncratic DILI can be classified, depending on the specific liver enzyme abnormality in each case, into hepatocellular, cholestatic, or mixed types [8]. Idiosyncratic DILI also can be classified as immune-mediated (allergic) and non-immune-mediated reactions. Immune-mediated idiosyncratic DILI typically occurs within 1 to 6 weeks following drug exposure and is associated with symptoms including fever, rash, eosinophilia, and autoantibodies, which may include ANA or ASMA. Non-immune-mediated idiosyncratic DILI, by contrast, does not manifest the symptoms listed above but presents later in terms of clinical manifestation. Identifying elevated liver enzymes, remains the hallmark for diagnosing DILI [11].

The mechanism of acetic acid-induced liver injury is not fully understood, but one hypothesis suggests that it may result from thrombosis and circulatory disturbances in the hepatic arteries and portal veins. Another hypothesis proposes that acetic acid causes direct toxic effects by damaging all layers of the mucosa and submucosa, ultimately reaching the portal and systemic circulation. Additionally, liver cell apoptosis could be considered another hypothesis, as many external agents, including toxins, are known to induce this form of cell death. Further research is needed to better understand the mechanism of liver damage following acetic acid ingestion [5].

The mortality outcome depends on the pattern of idiosyncratic DILI; mixed liver injury is associated with the best prognosis, while hepatocellular injury with jaundice carries the worst prognosis, with a mortality rate of 10% [12]. The crucial step in managing DILI is the immediate withdrawal of the drug. Indeed, in the majority of cases, this one intervention may be sufficient to ensure spontaneous resolution within days to weeks following cessation of drug intake, with further intervention usually not required. In some circumstances, it is not possible to anticipate the course of clinical and biochemical improvement as liver function may not return to normal despite drug withdrawal. This category of patients unquestionably needs regular follow-up and frequent blood investigations [10, 11].

## Conclusion

This case shows the rare possibility of vinegar-induced liver injury and emphasizes the importance of considering all possible etiologies in the diagnosis of DILI. Traditional substances should be considered when taking a medical history, as they can be major causes of liver damage. Our case also emphasizes the need for further research to better understand the mechanisms and risk factors associated with ACV-induced hepatotoxicity.
